# Supplementary material for: The causal relationship between the human gut microbiota and pyogenic arthritis: a Mendelian randomization study
Source: Front Cell Infect Microbiol. 2024 Nov 26;14:1452480. doi: 10.3389/fcimb.2024.1452480 (PMC11629706; doi:10.3389/fcimb.2024.1452480)
Supplement: Supplementary file 1 [file DataSheet1.zip › mendelian test/ebi-a-GCST90027618.csv_leave_one_out.pdf]

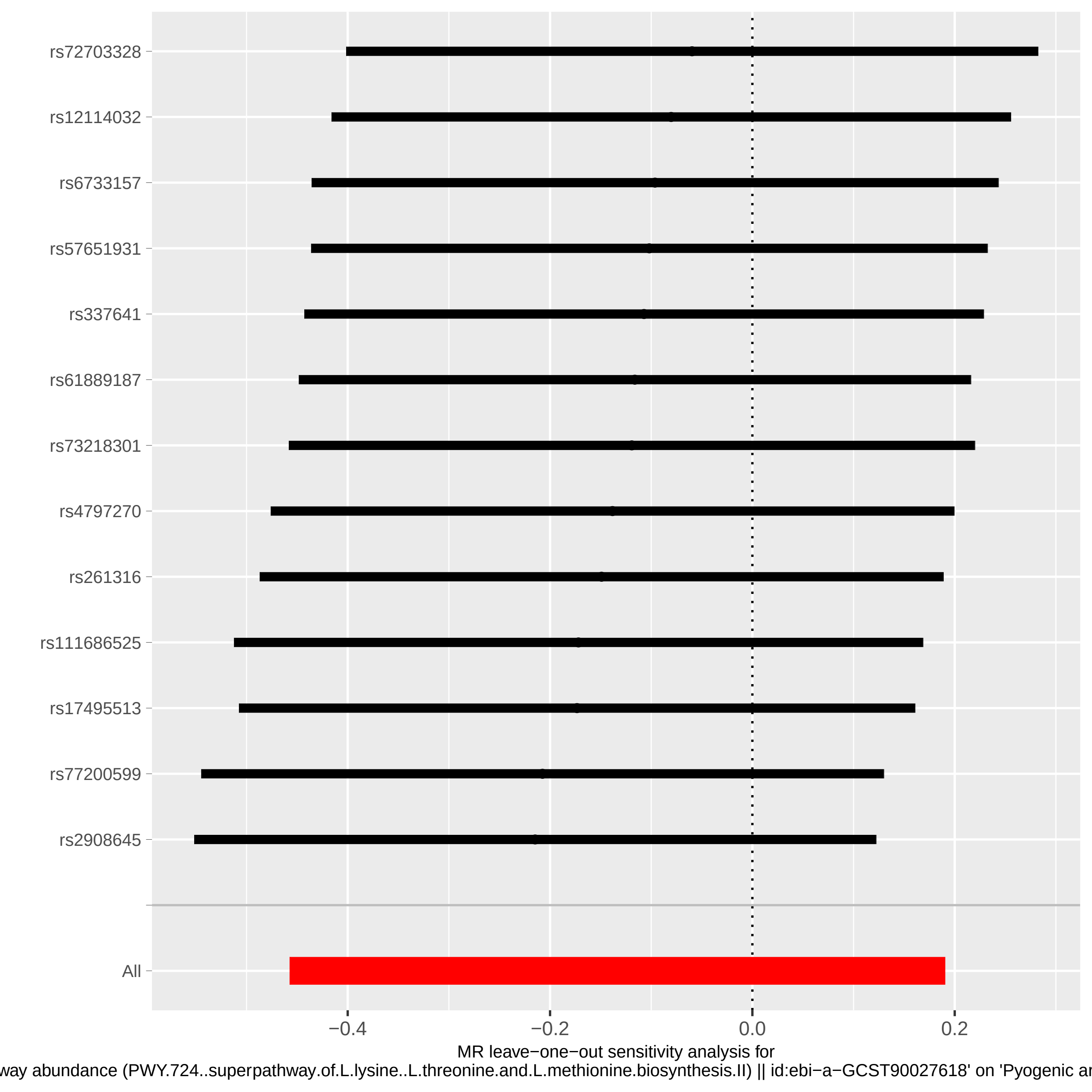
rs72703328
rs12114032
rs6733157
rs57651931
rs337641
rs61889187
rs73218301
rs4797270
rs261316
rs111686525
rs17495513
rs77200599
rs2908645
All
−0.4
−0.2
0.0
0.2
MR leave−one−out sensitivity analysis for
way abundance (PWY.724..superpathway.of.L.lysine..L.threonine.and.L.methionine.biosynthesis.II) || id:ebi−a−GCST90027618' on 'Pyogenic ar
